# Extrarenal multiorgan metastases of collecting duct carcinoma of the kidney: A case series

**DOI:** 10.1186/1752-1947-2-304

**Published:** 2008-09-17

**Authors:** Hisao Nakamura, Yasuyuki Kuirhara, Kazuhiko Matsushita, Akehide Sakai, Toshio Yamaguchi, Yasuo Nakajima

**Affiliations:** 1 Department of Radiology, Yokohama Sakae Kyousai Hospital, Yokohama, Japan; 2 Department of Radiology, St. Marianna University School of Medicine, Kawasaki, Kanagawa 216-8511, Japan; 3 Department of Pathology, Yokohama Sakae Kyousai Hospital, Yokohama, Japan; 4 Department of Urology, Yokohama Sakae Kyousai Hospital, Yokohama, Japan

## Abstract

**Introduction:**

Collecting duct carcinoma is a rare type of renal cell carcinoma. The primary is difficult to diagnose on imaging, and metastases are often present on initial presentation. Extensive multiorgan metastases can result in complex presentations that can be difficult to diagnose.

**Case presentation:**

We present two case reports of multiorgan metastases of collecting duct carcinoma that were autopsy confirmed. The first case was a 55-year-old man who presented with fever and abdominal pain. Abdominal computed tomography showed enlargement of the right kidney. Pyelonephritis was considered on the basis of laboratory test results and imaging findings. However, multiple cavitary lesions were found on routine chest radiography. These lesions were biopsied, resulting in a histological diagnosis of metastatic adenocarcinoma. A renal tumor was considered. Transitional cell carcinoma was suspected, which proved to be misdiagnosed and chemotherapy was given accordingly. However, this was not effective and the patient died after 2 months. Autopsy demonstrated the primary tumor to be collecting duct carcinoma, with metastases to lung, liver, spleen, bone marrow, right adrenal gland, and para-aortic lymph node. Computed tomography done while the patient was alive detected lung, liver, and para-aortic lymph node metastases. The second case was a 77-year-old man who presented with fever. Pyelonephritis was considered on the basis of the laboratory test results and imaging findings. Antibiotic therapy improved his symptoms and laboratory indicators of inflammation. One year later, he developed backache. Computed tomography revealed a progressively enlarging right renal lesion, multiple liver masses, enlargement of the para-aortic lymph nodes, and multiple osteoblastic and osteoclastic lesions. A renal tumor with multiple metastases was diagnosed. Chemotherapy was given without effect, and the patient died of cardiac failure 1 year later. Autopsy revealed a primary tumor of collecting duct carcinoma with metastases to the liver, right adrenal gland, right upper ureter, bone marrow, para-aortic and mediastinal lymph nodes, and bone.

**Conclusion:**

We present the radiological findings of lung, liver, lymph node, and bone metastases in two patients with collecting duct carcinoma.

## Introduction

Collecting duct carcinoma (CDC) is a rare type of renal cell carcinoma (RCC), accounting for 0.4% to 1.8% of all RCCs [1-3]. In general, this aggressive tumor is thought to have a dismal prognosis; early diagnosis appears to be the only factor that may result in prolonged survival [4,5]. Since patients with CDC often have metastases at the time of presentation, and computed tomography (CT) findings of the primary tumor can be difficult to interpret, it is important to be familiar with the radiological features of metastatic CDC based on autopsy-confirmed cases.

## Case presentation

### Case 1

**Figure 1:**
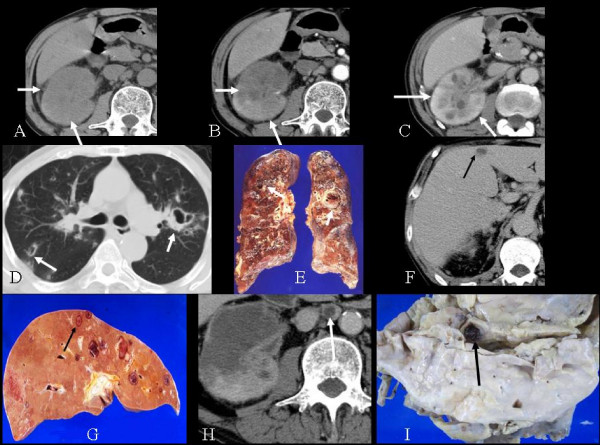
**A 55-year-old man with autopsy-confirmed collecting duct carcinoma and pulmonary, liver, and lymph node metastases**. A. Computed tomography scan shows an enlarged right kidney and an ill-defined mass (arrows). B. Early phase computed tomography reveals slow and heterogeneous enhancement of the lesion. C. Delayed phase computed tomography reveals slow and heterogeneous enhancement of the lesion. D. Computed tomography scan through the upper lung shows multiple cavitary lesions (arrows). E. Autopsy specimen reveals hemorrhagic nodules with central cavities (arrows) in both lungs. F. Enhanced computed tomography shows a low-attenuation area (arrow) with minimal enhancement in S4 of the liver. G. Autopsy specimen demonstrates a mass in S4 (arrow) with hemorrhagic and necrotic changes. H. Enhanced computed tomography shows a marginally enhanced nodule in the para-aortic area. I. Gross examination reveals a lymph node with central necrosis (arrow).

A 55-year-old man presented with fever and abdominal pain. Abdominal CT showed swelling of the right kidney and low attenuation areas (Figure 1A–C). Laboratory tests and renal CT imaging were suggestive of pyelonephritis. However, multiple cavitary lesions were also found on the routine chest radiography performed on admission. VATS (Video-Assisted Thoracic Surgery) lung biopsy confirmed the diagnosis of metastatic adenocarcinoma presumably from the kidney. Therefore, a renal tumor with multiple pulmonary metastases, was considered. At the time, transitional cell carcinoma (TCC) was suspected, which later proved to be a misdiagnosis, and chemotherapy with MVAC (methotrexate, vinblastine, adriamycin, and cisplatin) was given. However, the chemotherapy was not effective, and the patient died of respiratory failure 2 months later. On autopsy, the primary tumor was found to be a collecting duct carcinoma, and there were lung, liver, spleen, bone marrow, right adrenal gland, and para-aortic lymph node metastases. CT done while the patient was alive detected lung (Figure 1D, E), liver (Figure 1F, G), and para-aortic lymph node (Figure 1H, I) metastases.

### Case 2

**Figure 2:**
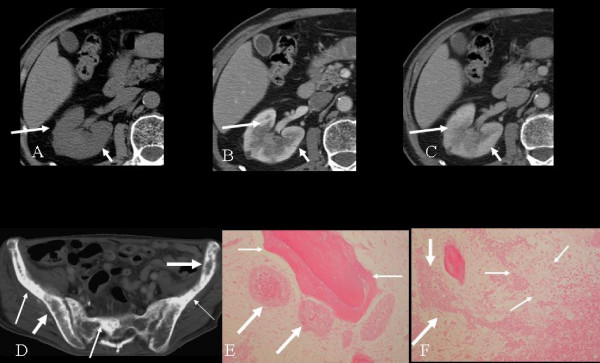
**A 70-year-old man with autopsy-confirmed collecting duct carcinoma and bony metastases**. A. Unenhanced computed tomography shows a poorly defined medullary tumor with infiltrative growth. The renal contour is intact. B. Early phase enhanced computed tomography scan reveals mild enhancement of the lesion. C. Delayed phase enhanced computed tomography scan reveals mild enhancement of the lesion. D. Computed tomography shows osteolytic (thin arrows) and osteosclerotic lesions (thick arrows) in the ilium and sacrum. E. Photomicrograph (×200, hematoxylin and eosin stain) demonstrates osteosclerotic changes (thin arrows) and tumor cells (thick arrows). F. Photomicrograph (×200, hematoxylin and eosin stain) shows osteolytic changes (thin arrows) and tumor cells (thick arrows).

A 77-year-old man was admitted to our department after developing a fever and backache. Based on CT findings (Figure 2A–C) and laboratory test results, pyelonephritis was initially suspected. Antibiotic therapy improved his symptoms and laboratory indicators of inflammation. One year later, he complained of backache. CT revealed a progressively enlarging renal lesion, multiple liver masses, enlargement of the para-aortic lymph nodes, and multiple osteoblastic and osteoclastic lesions (Figure 2D–F). A renal tumor with multiple metastases was suspected considering the clinical course and imaging findings retrospectively. At the time, TCC was suspected, which later proved to be a misdiagnosis. MVAC therapy was given without effect, and 1 year later the patient died of cardiac failure that was unrelated to the treatment. On autopsy, the primary tumor was found to be a collecting duct carcinoma, and liver, right adrenal gland, right upper ureter, bone marrow, para-aortic and mediastinal lymph node, and bone metastases were found.

## Discussion

CDC is an uncommon yet distinct epithelial neoplasm of the kidney [6]. Unlike the more common types of renal cell carcinoma that arise from the convoluted tubules of the renal cortex, CDC is derived from the renal medulla, possibly from the distal collecting duct of Bellini.

Characteristic imaging findings of CDC are not well delineated because only case reports or studies involving small numbers of patients have been published to date. Fukuya *et al*. [7] reported five cases with small CDC tumors, all measuring between 3 and 4.5 cm: all five lesions were centered in the renal medulla; four of them protruded into the central sinus; and none showed exophytic growth. Pickhardt *et al*. [8] reported similar results for tumors less than 5 cm in diameter, though the majority of tumors in that series was larger than 5 cm; in large tumors, the central area was overshadowed by an exophytic or expansile component, and it was difficult to recognize the medullary origin. Furthermore, patients with advanced CDC frequently have fever, and invasive CDC sometimes resembles and is associated with severe pyelonephritis or xanthogranulomatous pyelonephritis. Thus, when the CDC is large and invasive, it is difficult to make the correct diagnosis based on imaging alone.

Up to 40% of CDC patients have metastatic disease at the time of presentation [6]. In cases that present with metastatic CDC, radical nephrectomy alone does not appear to be effective due to technical difficulties related to surgery and a low survival rate [9]. Our cases had cavitary pulmonary metastases and marginally enhanced lesions with necrosis in the liver and para-aortic lymph nodes. These findings represent necrotic changes that are common in both the primary CDC tumor and its metastases [6] and reflect the aggressive nature of the disease.

One of our cases had bone metastases that exhibited both osteolytic and osteoblastic features. This pattern of bony metastases was also observed in a recent report [10].

## Conclusion

When extensive multiorgan metastases with necrotic changes are seen along with aggressive involvement of the kidney, the differential diagnosis of the primary tumor should include collecting duct carcinoma.

## Consent

Written informed consent for publication of these case reports and any accompanying images was obtained from the patients in both cases. A copy of the written consent is available for review by the Editor-in-Chief of this journal.

## Competing interests

The authors declare that they have no competing interests.

## Authors' contributions

HN conception and acquisition of data. YK design and analysis of data and drafting the manuscript. MK acquisition and interpretation of data. AS interpretation of data and drafting the manuscript. TY drafting and revising the manuscript. YN revising and final approval of the manuscript
